# Personal and Ambient Air Pollution is Associated with Increased Exhaled Nitric Oxide in Children with Asthma

**DOI:** 10.1289/ehp.9141

**Published:** 2006-07-11

**Authors:** Ralph J. Delfino, Norbert Staimer, Dan Gillen, Thomas Tjoa, Constantinos Sioutas, Kochy Fung, Steven C. George, Michael T. Kleinman

**Affiliations:** 1 Epidemiology Division, Department of Medicine, School of Medicine, University of California, Irvine, Irvine, California, USA; 2 Department of Statistics, University of California, Irvine, Irvine, California, USA; 3 Department of Civil and Environmental Engineering, School of Engineering, University of Southern California, Los Angeles, California, USA; 4 AtmAA, Inc., Calabasas, California, USA; 5 Department of Biomedical Engineering, University of California, Irvine, Irvine, California, USA; 6 Department of Community and Environmental Medicine, School of Medicine, University of California, Irvine, Irvine, California, USA

**Keywords:** asthma, epidemiology, exhaled nitric oxide, longitudinal data analysis, nitrogen dioxide, ozone, panel study, particulate air pollution

## Abstract

**Background:**

Research has shown associations between pediatric asthma outcomes and airborne particulate matter (PM). The importance of particle components remains to be determined.

**Methods:**

We followed a panel of 45 schoolchildren with persistent asthma living in Southern California. Subjects were monitored over 10 days with offline fractional exhaled nitric oxide (Fe_NO_), a biomarker of airway inflammation. Personal active sampler exposures included continuous particulate matter < 2.5 μm in aerodynamic diameter (PM_2.5_), 24-hr PM_2.5_ elemental and organic carbon (EC, OC), and 24-hr nitrogen dioxide. Ambient exposures included PM_2.5_, PM_2.5_ EC and OC, and NO_2_. Data were analyzed with mixed models controlling for personal temperature, humidity and 10-day period.

**Results:**

The strongest positive associations were between Fe_NO_ and 2-day average pollutant concentrations. Per interquartile range pollutant increase, these were: for 24 μg/m^3^ personal PM_2.5_, 1.1 ppb Fe_NO_ [95% confidence interval (CI), 0.1–1.9]; for 0.6 μg/m^3^ personal EC, 0.7 ppb Fe_NO_ (95% CI, 0.3–1.1); for 17 ppb personal NO_2_, 1.6 ppb Fe_NO_ (95% CI, 0.4–2.8). Larger associations were found for ambient EC and smaller associations for ambient NO_2_. Ambient PM_2.5_ and personal and ambient OC were significant only in subjects taking inhaled corticosteroids (ICS) alone. Subjects taking both ICS and antileukotrienes showed no significant associations. Distributed lag models showed personal PM_2.5_ in the preceding 5 hr was associated with Fe_NO_. In two-pollutant models, the most robust associations were for personal and ambient EC and NO_2_, and for personal but not ambient PM_2.5_.

**Conclusion:**

PM associations with airway inflammation in asthmatics may be missed using ambient particle mass, which may not sufficiently represent causal pollutant components from fossil fuel combustion.

Epidemiologic studies have shown associations between asthma outcomes and criteria air pollutants regulated by the U.S. Environmental Protection Agency (EPA), such as nitrogen dioxide and mass concentrations of particulate matter (PM) < 2.5 μm in aerodynamic diameter (PM_2.5_). It is unclear whether these associations for pollutant gases and particles are independent of each other or are attributable to toxic air pollutants not routinely monitored, such as combustion-related organic compounds (Delfino 2002). Growing experimental evidence suggests that PM organic components, including polycyclic aromatic hydrocarbons (PAHs), have adjuvant effects on cytokine-mediated airway inflammation, at least partly through exposure to redox active chemicals and resultant oxidative stress (Li et al. 2003). The relevance of these findings to people with asthma can be enhanced with an assessment of the impact of personal air pollutant exposures on acute changes in asthma outcomes. Children with asthma at greatest risk of exacerbations from air pollutants likely include those with persistent asthma symptoms.

Airway inflammation is a hallmark of asthma. The fractional concentration of nitric oxide in exhaled air (Fe_NO_) is a noninvasive biomarker of airway inflammation and is higher in subjects with poorly controlled asthma (Jones et al. 2001). Therefore, Fe_NO_ is potentially useful in epidemiologic field research to evaluate the impacts of air pollution on the inflammatory state of airways in children with asthma. NO is produced endogenously in the airways from l-arginine by NO synthetase. There are two constitutive isoforms and an inducible isoform involved in airway inflammation. Inflammatory cytokines (e.g., tumor necrosis factor-α) increase the expression of inducible NO synthetase in airway epithelium (Zeidler et al. 2004). There is extensive evidence that Fe_NO_ is elevated in patients with untreated asthma and decreases with corticosteroid treatment (Zeidler et al. 2004; Zeiger et al. 2006). There is also evidence that Fe_NO_ correlates with eosinophilic inflammation in asthmatic patients, especially in atopic subjects (Zeidler et al. 2004). After withdrawal of inhaled steroids, Fe_NO_ accurately predicts loss of asthma control (positive predictive value 80–90%) just as well as either eosinophils from induced sputum or bronchial hyperresponsiveness (Jones et al. 2001).

We examined the relationship of daily Fe_NO_ with personal and ambient air pollution exposures in 45 schoolchildren with asthma. We hypothesized that airway inflammation in asthmatic children as measured by Fe_NO_ would be positively associated with personal exposure to PM_2.5_, elemental and organic carbon fractions of PM_2.5_ (EC and OC), and NO_2_. We further aimed to determine whether EC, OC, or NO_2_ is more strongly and precisely associated with Fe_NO_ than PM_2.5_ mass. By measuring personal particle exposures, we aimed to confirm and clarify our earlier findings that associations of asthma symptoms with ambient mass concentrations of PM < 10 μm in aerodynamic diameter (PM_10_) were better explained by EC and OC fractions of ambient PM_10_ (Delfino et al. 2003). In that panel study, 20 Hispanic children with asthma were followed daily over 24 days in a small Los Angeles (LA) community surrounded by freeways. A positive association of asthma symptoms with PM_10_ was reduced to unity when co-regressed with ambient EC or OC fractions of PM_10_, but larger positive associations of EC and OC with symptoms remained stable. This finding suggests that combustion-related organic compounds such as those in diesel exhaust are causally related to acute asthma. EC and OC may have represented particle components (e.g., PAHs) and key sources that are believed to play a causal role (Li et al. 2003). As crude markers of pollutant sources, EC primarily represents products of fossil fuel combustion in the LA Basin, especially diesel exhaust, and OC represents both fossil fuel combustion and secondary organic aerosols (Fine et al. 2004; Schauer et al. 2002). Similarly, NO_2_ is strongly influenced by local traffic density (Kim et al. 2004; Roorda-Knape et al. 1999), and it may serve as a surrogate for particulate air pollutants more causally related to acute changes in asthma morbidity. Therefore, we also aimed to determine whether personal or ambient PM_2.5_, EC, or OC exposures interact with or confound personal or ambient NO_2_ in relation to Fe_NO_.

## Materials and Methods

### Design and population

This panel study involves repeated daily measurements of health outcomes and exposures in children with asthma. It was conducted in urban regions of Southern California with high levels of air pollution, primarily from mobile sources of fossil fuel combustion. The first region was in Riverside, California, where subjects were followed from August through mid-December 2003. This is a downwind smog receptor site just inland from LA County. The second region was in Whittier, California, in eastern LA County, where subjects were followed from July through November 2004. Data reported in this article are from an intensive phase of study over 10-day periods. This involved having subjects wear an active personal monitor to measure exposure to air pollutants, and collection of an offline measurement of Fe_NO_ at the end of each 24 hr of personal sampling. Data for both Fe_NO_ and personal air pollutant exposures were collected from 48 asthmatic children (16 from Riverside) over twelve 10-day periods, four subjects per period. Subjects were followed daily in their homes to check the validity of exposure and outcome data and to ensure compliance. Each subject completed a diary on personal digital assistants every two waking hours. Questions addressed preventive medication use and respiratory infections. Missed prompts were mitigated with paper diaries and daily technician-administered questionnaires.

The institutional review board of the University of California, Irvine (UCI) approved the study protocol. Informed written assent was obtained from subjects and consent was obtained from one of their legal guardians. Subjects who were 9–18 years of age were recruited through notification of parents by public schools. We targeted subjects with persistent asthma having a history in the previous 12 months of exacerbations of asthma symptoms requiring the use of as-needed bronchodilators ≥ 2 days/week, or taking oral or inhaled anti-inflammatory medications, regardless of recent symptom frequency. Finally, subjects were recruited with < 80% predicted forced expiratory volume in 1 sec (FEV_1_) from baseline spirometry. Subjects were ineligible if they smoked or if someone smoked in the subject’s home.

### Measurement of exhaled NO

We measured exhaled NO using recommended procedures [American Thoracic Society/European Respiratory Society (ATS/ERS) 2005], with modifications suggested by Linn et al. (2004) and Kroesbergen et al. (1999). The method involved collection of exhalate into receptacles for later chemical analysis. Subjects were asked to refrain from performing spirometry, exercise, and food or beverage intake 1 hr before sample collection. Subjects inhaled orally to total lung capacity and then immediately performed a slow vital capacity maneuver into an offline apparatus attached to a nonreactive 1.5-L Mylar reservoir bag (Ionics Inc., Boulder, CO). The flow rate was to be 100 mL/sec at all times by maintaining pressure at 19 cm H_2_O, which is suitable for children with asthma (Kroesbergen et al. 1999). Approximately 200 mL of dead-space air was vented before the bag sample was collected, to reduce contamination from the upper airways (Jobsis et al. 2001). To control for inspired ambient NO, an NO/NO_2_ chemisorbent filter was placed at the air intake of the offline apparatus, and subjects breathed through it for 15 sec (≥ 2 tidal breaths) before sampling. Two breath samples were collected to assess reliability. The 10 daily collections were done at the same time of day for each subject. An indoor air sample was collected to assess influence of indoor NO on Fe_NO_ despite use of the NO scrubbing filter (ATS/ERS 2005). Mylar bags were sealed, refrigerated at 6°C, and analyzed within 20 hr for Fe_NO_ concentration with a chemiluminescence NO analyzer (NOA 280i Sievers; GE Analytical Instruments, Boulder, CO). We retained for analysis the mean of Fe_NO_ pairs where differences were ≤ 3 ppb or ≤ 10% of the larger reading.

### Exposures

Our personal exposure monitor was constructed to collect 24-hr samples of personal PM_2.5_, EC and OC fractions of PM_2.5_, NO_2_, temperature, and relative humidity (RH). Each subject wore the monitor during waking hours in a backpack. Personal PM_2.5_ monitors operated at 4 L/min with the sampling inlet near the breathing zone. One-minute average PM_2.5_ was measured using an integrated nephelometer, the personal DataRAM model 1200 (pDR; MIE Inc., Bedford, MA). Compliance (motion), RH, and temperature loggers recorded at 1-min intervals (Onset Computer Corp., Pocasset, MA). Data were downloaded and checked for quality and compliance daily. pDR data were adjusted for personal RH (Chakrabarti et al. 2004). The pDR configuration also included a filter cartridge for EC and OC. A 2.5-μm sharp-cut cyclone was attached upstream of the pDR, and PM_2.5_ was collected on 37-mm quartz filters (Whatman Inc., Florham Park, NJ). The personal PM_2.5_ monitor was validated by our group in a previous report and it is described in detail there (Chakrabarti et al. 2004).

We developed an active sampling system for NO_2_ that ran in parallel with the pDR. It uses a miniaturized diaphragm pump (Virtual Industry VMP1625, Colorado Springs, CO) run at 0.1 L/min, and triethanolamine-treated molecular sieve sorbent tubes as a collection medium (SKC West Inc., Fullerton, CA). NO_2_ measurements were based on National Institute for Occupational Safety and Health method 6014 (National Institute for Occupational Safety and Health 1994). This personal NO_2_ exposure monitor was validated by our group in a previous report and it is described in detail there (Staimer et al. 2005).

Central-site ambient exposures included 24-hr PM_2.5_, PM_10_, and PM_2.5_ EC and OC collected from early evening to early evening with Harvard Impactors (Air Diagnostics and Engineering, Inc., Naples, ME) using standard procedures. After sample collection on quartz filters, particulate carbon was speciated into OC and EC using the thermal manganese dioxide oxidation technique (for details, see Fung et al. 2002). EC and OC are expressed as amount of total carbon per sample. Central-site data also included hourly ozone, NO_2_, and CO measured by the South Coast Air Quality Management District. In Riverside, the district site was centrally located. In Whittier, we constructed a central site at a subject home elevated on a hill. Data for O_3_, NO_2_, and CO came from two district sites at opposite ends of the Whittier study region (La Habra and Pico Rivera). Hourly concentrations for the two stations were averaged.

### Statistical methods

We used linear mixed-effects models (Verbeke and Molenberghs 2001) to estimate the association between each air pollutant and Fe_NO_. Because the data represent repeated measures on individuals over time, correlation among outcomes was present. We assumed a two-stage hierarchical model with random effects at the subject level, nested within exposure run. Empirical variograms (graphical measures of the correlation between observations as a function of time) showed an autoregressive-1 correlation structure adequately described the observed variability. All exposures were mean-centered by individual to obtain comparability from one participant location to another (Sheppard et al. 2005). We investigated exposures preceding the Fe_NO_ measurement including the last 24 hr (lag 0), the average of the 25th through 48th hour preceding the Fe_NO_ measurement (lag 1), and a cumulative 2-day moving average (2-day MA). Results are expressed as ppb change in Fe_NO_ per interquartile range (IQR) increase in pollutant.

Given their potential for confounding associations, we decided *a priori* that personal temperature, personal RH, and 10-day exposure run should be adjusted for in all models. We also tested potential confounding by respiratory infections, region of study, sex, cumulative daily use of as-needed β-agonist inhalers, and weekend. None of these variables confounded associations, and there was no air pollutant interaction with sex or respiratory infection (only 13 person-days reported). Secondary analyses examining potential effect modification by medication use were also conducted. Residual diagnostics were conducted for all models to investigate the presence of influential data points and deviations from assumed functional form. No data points were removed for influence. As a validity check to the likelihood assumptions made by the linear mixed-effects model, regression models using generalized estimating equations (Diggle et al. 2002) in combination with robust standard error estimates were also fit. No qualitative differences in our study results were found.

To investigate the lag effect of hourly personal PM_2.5_ on Fe_NO_, we used a fourth-order polynomial distributed lag mixed-effects model (Schwartz 2000). An autoregressive correlation structure was assumed and supported by residual diagnostics. Distributed lag models stratifying on anti-inflammatory medication use were also fit.

Between-pollutant confounding was tested with two-pollutant regression models. We compared the change in regression parameters from single- to two-pollutant models for the same subset of nonmissing person-days for both pollutants. Interaction between pollutants was tested first in a regression equation with the two pollutant variables and their product term as predictors.

## Results

### Descriptive subject data

The Riverside panel included eight 10-day runs with four subjects per run. However, the Sievers NO Analyzer malfunctioned after the third day of run 5. The analyzer was replaced by a new one, but not until after the end of Riverside run 8. In addition, personal samplers malfunctioned during most of the entire first run for three subjects. This was resolved before the next run. Therefore, we retained for analysis data from the first four 10-day runs in Riverside involving 13 subjects. Data were collected for all eight 10-day runs in Whittier for 32 subjects and combined with data from Riverside. Table 1 shows characteristics of the 45 subjects.

Of 446 daily pairs of Fe_NO_ samples, we found that 372 pairs (83%) were reliable by our criteria (≤ 3 ppb NO or ≤ 10% difference). In the Supplemental Material (http://www.ehponline.org/docs/2006/9141/suppl.pdf), we present analyses of relationships of Fe_NO_ with the quality of maneuver and noncompliance with pretesting spirometry, exercise, food, and beverages. Controlling for maneuver quality and noncompliance factors did not confound the associations of Fe_NO_ with air pollutants. In addition, there was no relationship between indoor NO and acceptable Fe_NO_ pairs (slope 0.04 ± 0.03, *p* = 0.21), and indoor NO concentration did not influence associations of air pollutant exposures with Fe_NO_.

The distribution of Fe_NO_ concentrations by region and medication use is shown in Table 2. Subjects in Whittier had nonsignificantly higher Fe_NO_ than did Riverside subjects. As confirmed in daily diaries and by research staff, 14 subjects were not taking anti-inflammatory controller medications and 31 were [inhaled corticosteroids (ICS) and antileukotrienes (leukotriene receptor antagonists zafirlukast and montelukast)]. Subjects not taking any anti-inflammatory medication had higher mean Fe_NO_, consistent with expectations (Zeidler et al. 2004). There was no difference within the anti-inflammatory medication group by use of antileukotrienes.

### Descriptive exposure data

Table 3 shows descriptive data for exposures by region. Central-site particle mass, EC, OC, NO_2_, and O_3_ concentrations were higher in Riverside because this warmer receptor region is located downwind of major urban sources in LA, adding to local sources of these air pollutants. Despite lower ambient concentrations, personal EC, OC, and NO_2_ were somewhat higher in Whittier than in Riverside, perhaps reflecting proximity of subjects to densely populated LA areas having a higher local traffic impact. Maximum 1-hr personal PM_2.5_ ranged up to 573 μg/m^3^.

Table 4 shows the between-pollutant correlations. Correlations of personal PM_2.5_ with personal EC, OC, and NO_2_ were significant but small, and not much different from correlations with ambient data. Personal EC and OC were not correlated with ambient EC and OC but were correlated with ambient NO_2_. Personal PM_2.5_ was moderately correlated with ambient PM_2.5_ (*r* = 0.64), and personal NO_2_ was moderately correlated with ambient NO_2_ (*r* = 0.46). Ambient exposures were all moderately correlated with each other.

### Regression models for personal as compared with central-site air pollutants

We found positive associations of Fe_NO_ with increasing personal PM_2.5_ mass, EC, and NO_2_, and central-site EC, OC, and NO_2_ (Table 5). The most robust associations [largest coefficient and/or tightest 95% confidence interval (CI)] were between Fe_NO_ and 2-day average exposures for all regression models. Exhaled NO was associated with personal PM_2.5_, but was not associated with ambient PM_2.5_ (*p* > 0.22) or ambient PM_10_ (not shown). Except for lag 0 EC, the associations of Fe_NO_ with EC and OC were stronger for central-site than for personal measurements, but 95% CIs overlapped extensively and lower bounds for all of the OC models were negative. The association of Fe_NO_ with personal and central-site 2-day average NO_2_ was similar. There was little difference in associations using 8-hr maximum central-site NO_2_ (not shown). Ambient CO and O_3_ were not associated with Fe_NO_ (not shown). Including 74 person-days of sampling with the means of the unreliable Fe_NO_ pairs did not alter strengths of association but SEs increased as expected. The exception was for 2-day MA personal PM_2.5_, where the parameter estimate decreased from 0.041 ± 0.019 to 0.030 ± 0.020.

### Differences in association by medication use

Table 6 shows results for models stratified by medication use. We found associations with personal NO_2_, and ambient EC, OC, and NO_2_ were stronger and more significant in the group on anti-inflammatory medications than in the group not on anti-inflammatory medications. However, product terms for these pollutants by a medication group indicator were not significant at *p* < 0.1. The anti-inflammatory medication group was next split into subjects only taking ICS and subjects taking antileukotrienes with or without ICS. Results suggest effect modification by antileukotrienes combined with ICS. Among the group taking ICS but not antileukotrienes, all air pollutants were significantly associated with Fe_NO_, or nearly so in the case of ambient PM_10_. Personal and ambient OC and ambient PM_2.5_ were significantly associated with Fe_NO_ only in the group taking ICS but not antileukotrienes. Product terms for controller medication group by personal PM_2.5_ and by ambient PM_2.5_ and PM_10_ were all significant. We found no meaningful change in the null results dropping two of the 12 subjects taking antileukotrienes who were not also taking ICS.

### Distributed lag models for hourly personal PM_2.5_

**Figure 1:**
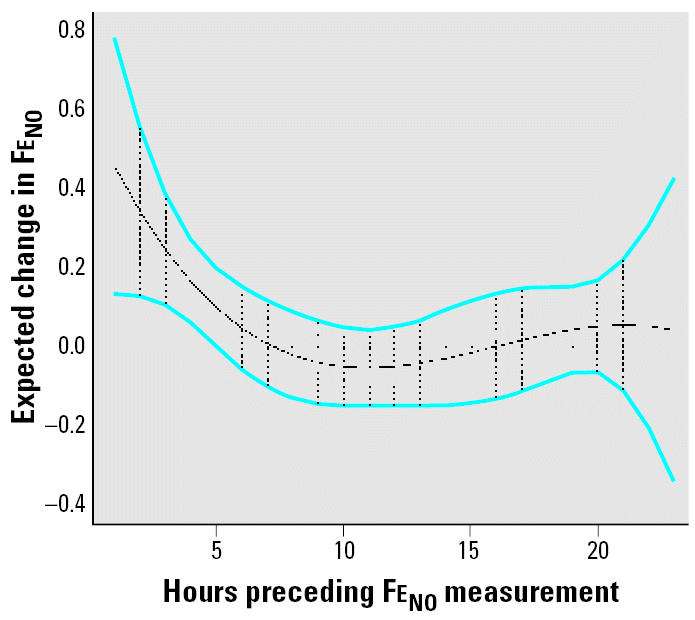
Estimated lag effect of hourly personal PM_2.5_ on Fe_NO_. Estimates are based on a 4th-degree linear mixed-effects polynomial distributed lag model with AR(1) correlation structure. Expected changes in Fe_NO_ correspond to a 1-IQR (24 μg/m^3^) change in PM_2.5_. Blue bands indicate pointwise 95% CIs. Vertical dashes represent hourly measurements. All estimates are adjusted for personal temperature and relative humidity.

Figure 1 shows that Fe_NO_ in all 45 subjects is acutely associated with PM_2.5_ exposure in the 5 hr preceding measurement, with the lower 95% confidence bound crossing zero after lag 5 and coefficients remaining near zero after that. Beyond 24 hr, we found no significant association between PM_2.5_ and Fe_NO_, partly because of the high variability resulting from such large lag times. The associations are stronger the closer the personal PM_2.5_ measurement is to the Fe_NO_ measurement. At lag 0 hr, Fe_NO_ was estimated to increase 0.46 ppb (95% CI, 0.13–0.78) per one-IQR increase in personal PM_2.5_ (24 μg/m^3^).

**Figure 2:**
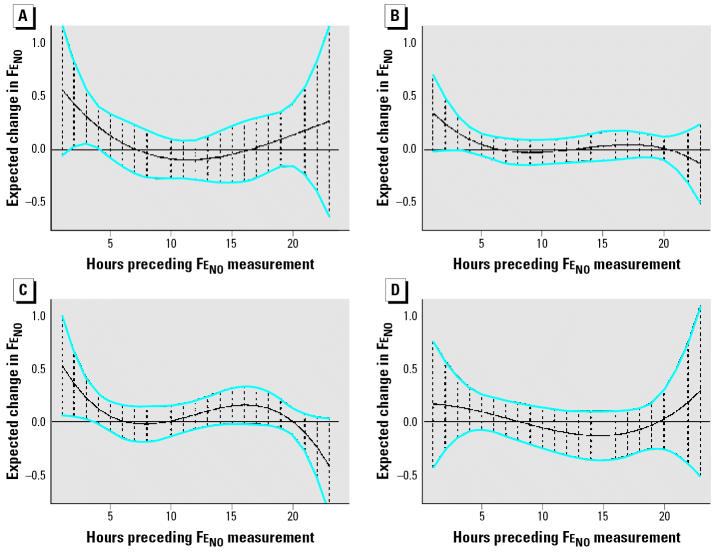
Estimated lag effect of hourly personal PM_2.5_ on Fe_NO_ by use of medications. (*A*) No anti-inflammatory medications. (*B*) Anti-inflammatory medications. (*C*) Inhaled corticosteroids. (*D*) Antileukotrienes and inhaled corticosteroids. Estimates are based on a 4th-degree linear mixed-effects polynomial distributed lag model with AR(1) correlation structure. Expected changes in Fe_NO_ correspond to a 1-IQR (24 μg/m^3^) change in PM_2.5_. Blue bands indicate pointwise 95% CIs. Vertical dashes represent hourly measurements. All estimates are adjusted for personal temperature and relative humidity.

Figure 2 shows that associations of Fe_NO_ with lag 0–5 hr personal PM_2.5_ were moderately stronger in the group not on anti-inflammatory medications. At lag 0 hr, Fe_NO_ increased 0.57 ppb (95% CI, −0.06 to 1.20) per IQR increase in personal PM_2.5_ (24 μg/m^3^) among subjects not taking anti-inflammatory medications (Figure 2A), compared to 0.35 ppb (95% CI, −0.02 to 0.71) among subjects taking anti-inflammatory medications (Figure 2B). When the medication group was further divided based on use of antileukotrienes, we found an early association between PM_2.5_ and Fe_NO_ among patients taking only inhaled corticosteroids (Figure 2C). At lag 0 hr, Fe_NO_ increased by 0.53 ppb (95% CI, 0.06–1.00), but there was no association among patients on antileukotrienes (Figure 2D), consistent with results using the 2-day MA.

We also tested models for the relationship between Fe_NO_ and 1-hr and 8-hr maximum personal PM_2.5_ in the previous 24 hr (not shown). We found that for all 45 subjects, an IQR increase of 73 μg/m^3^ 1-hr maximum personal PM_2.5_ was associated with a 0.60-ppb increase in Fe_NO_ (95% CI, 0.14–1.05). Eight-hour maximum personal PM_2.5_ (37 μg/m^3^) was similarly associated with Fe_NO_ (0.54 ppb; 95% CI, 0.09–1.16). We found a stronger association between Fe_NO_ and 1-hr maximum personal PM_2.5_ in subjects not taking anti-inflammatory medications (1.36 ppb, 95% CI, 0.07–2.79) compared with those taking them (0.46 ppb; 95% CI, 0.05–0.87), but the difference was not significant (*p* = 0.14).

### Two-pollutant models

**Figure 3:**
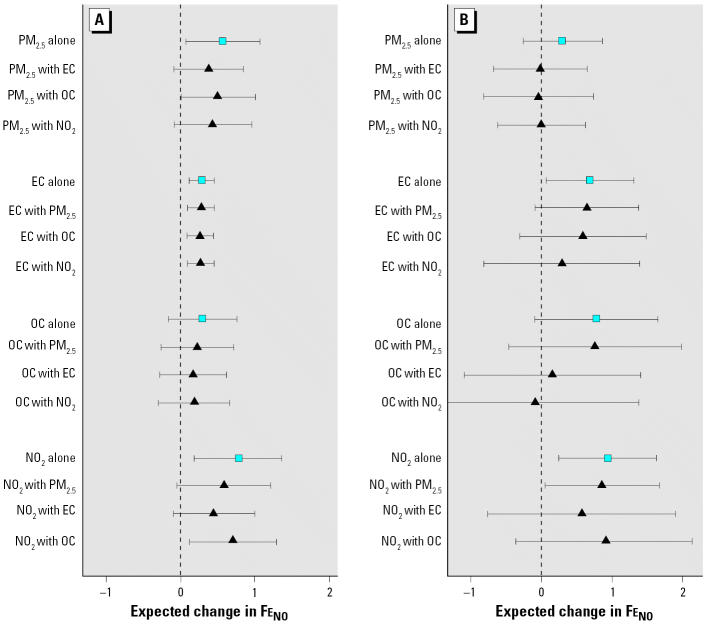
One- and two-pollutant models for change in Fe_NO_ using 2-day MA personal (*A*) and central-site (*B*) pollutant measurements. Squares: single-pollutant model; triangles: two-pollutants models. Expected change in Fe_NO_ is per IQR in the pollutant of interest with 95% CIs. All estimates are adjusted for personal temperature and relative humidity.

The only significant interaction between air pollutants tested in the two-pollutant models was a positive interaction between personal PM_2.5_ and personal OC (*p* = 0.003). Figure 3 shows models for personal exposures and separately for ambient exposures. For personal two-pollutant models, the most robust association was for EC, followed by NO_2_, PM_2.5_, and then OC. For two-pollutant models using ambient data, we found CIs widened for both pollutants, partly due to their correlation (Table 3). Co-regression of ambient PM_2.5_ with ambient EC, OC, or NO_2_ led to a large reduction in the parameter for PM_2.5_ to near zero, but little change in estimates of association for EC, OC, or NO_2_. Ambient OC was markedly confounded by both NO_2_ and EC. Overall, the ambient two-pollutant models show the most robust association for NO_2_ and EC.

To assess whether the association of Fe_NO_ in all 45 subjects with ambient EC or NO_2_ was independent of personal EC or NO_2_, we co-regressed two-day average ambient with personal EC (not correlated; Table 4), and personal with ambient NO_2_ (moderately correlated). Associations for personal and central-site EC were completely stable compared with single-pollutant models (Table 5) [0.73 ppb (95% CI, 0.30–1.15) and 1.37 ppb (95% CI, 0.13–2.60), respectively]. Personal NO_2_ completely confounded ambient NO_2_ [1.46 ppb (95% CI, −0.24 to 3.16) and 0.19 ppb (95% CI, −1.24 to 1.62), respectively].

To assess whether the positive association of Fe_NO_ in 19 subjects on ICS with ambient PM_2.5_ or OC was independent of personal PM_2.5_ or OC, we co-regressed ambient with personal exposures for the same pollutant. The association with ambient PM_2.5_ was completely confounded by personal PM_2.5_, which showed nearly the same positive association with Fe_NO_ as the single-pollutant model in Table 6. The between-pollutant correlation was 0.53. Similarly, the association with ambient OC was halved, whereas personal OC was minimally reduced by 13%. The between-pollutant correlation was nonsignificant. The isolated association of ambient PM_2.5_ with Fe_NO_ in this group was independent of ambient EC, OC, and NO_2_.

## Discussion

Positive associations were found for Fe_NO_ in relation to personal and ambient air pollutants, with evidence from the multiple-pollutant approach that traffic-related sources of air pollutants underlie the findings. Although the estimates of effect were small (≤ 2.5 ppb Fe_NO_), inasmuch as Fe_NO_ is a marker of airway inflammation, this would suggest that air pollution increases inflammation. We cannot say whether the chosen estimate of effect by the interquartile range increase in air pollution is clinically relevant or not.

Other studies have found associations of ambient air pollutants with Fe_NO_ in elderly adults with asthma (Jansen et al. 2005), adults with cardiac disease (Adamkiewicz et al. 2004), healthy adults (van Amsterdam et al. 1999), and general populations of schoolchildren (Fischer et al. 2002; Steerenberg et al. 2001, 2003). Our findings are most consistent with a panel study of 19 asthmatic children in Seattle, Washington (Koenig et al. 2003). In nine children not taking ICS, they found an approximately 4-ppb increase in Fe_NO_ per 10-μg/m^3^ lag 0 personal, indoor and outdoor home, and ambient PM_2.5_. In a follow-up report of the Seattle panel study, Koenig et al. (2005) found that the estimated ambient-generated fraction of personal PM_2.5_ exposure was positively associated with Fe_NO_, but not the estimated indoor-generated fraction.

Results of models using distributed hourly lags and daily average personal PM_2.5_ point to possible cumulative and lag effects (48-hr mean and 1-day lag) and more immediate effects (previous 5 hr) on Fe_NO_. Findings in other panel studies also support a rapid effect of recent hourly PM_2.5_ on biomarkers of inflammation in children with asthma, including the aforementioned Seattle study using Fe_NO_ (Mar et al. 2005), and a Denver, Colorado, study using urinary leukotriene E_4_ (Rabinovitch et al. 2006). This may reflect acute-phase inflammation from an early release of mediators by mast and other cells, followed by a late-phase response peaking a few hours later and characterized by lymphocyte activation and infiltration (Hamid et al. 2003). However, experimental data are needed to provide evidence that is more definitive for a delayed (or cumulative) effect from multiday exposures.

The generally more robust associations for personal than for central-site PM_2.5_ exposures suggest that nondifferential exposure misclassification leads to attenuation of the particle association and increased variance. This is graphically demonstrated in Figure 3. A coherent contrast between personal and ambient PM_2.5_ using FEV_1_ as an outcome was found in our previous asthma panel study (Delfino et al. 2004). In addition, the present associations of Fe_NO_ with outdoor ambient EC and OC but not ambient PM_2.5_ suggest that PM associations may be missed using total particle mass measurements alone, coherent with our previous findings for ambient EC, OC, and symptoms in asthmatic children in Los Angeles (Delfino et al. 2003). Furthermore, the present significant associations of Fe_NO_ with both personal and ambient EC and NO_2_ suggest that despite potential misclassification of personal exposure, ambient data linked to combustion sources represents casual pollutant components.

Both personal and ambient two-pollutant models showed that EC and NO_2_ had the most robust associations against inclusion of other pollutants. However, in contrast to ambient data, personal exposure models showed little to no confounding of personal PM_2.5_ by EC or NO_2_, suggesting that other causal factors in personal PM exposures are not represented by EC or NO_2_. Both EC and NO_2_ originate in the study region largely from traffic-related sources, which have high spatial variability (Sioutas et al. 2005), thereby creating highly variable personal exposures to EC and NO_2_ in the present study population. Indoor concentrations represent the collective contributions of local traffic and other outdoor sources, as well as indoor sources and activities, such as cooking. Similarly, outdoor OC is dominated by vehicular emissions and secondary organics, whereas indoor OC includes contributions from cooking and other sources of nontoxic semivolatile OC, in addition to outdoor OC that penetrates indoors. This, plus imprecision of the personal monitor for OC, may have increased misclassification of personal OC exposure and weakened associations. Personal PM_2.5_, on the other hand, likely represents a variety of indoor particle components that can induce asthma, including allergens and endotoxin (Rabinovitch et al. 2005). It also represents ambient PM_2.5_ given the moderate correlation between ambient and personal PM_2.5_ (*R* = 0.63, *p* < 0.001). Interaction between personal PM_2.5_ and OC suggests a possible interactive particle effect.

To assess further the potential contribution of ambient air pollution to associations of Fe_NO_ with personal exposures, we tested two-pollutant models with the same ambient and personal pollutant. Personal and ambient EC were independently associated with Fe_NO_, whereas personal NO_2_ confounded the association of ambient NO_2_ with Fe_NO_. Lack of correlation between personal and ambient EC suggests that different sources contribute to the concentrations. The moderate personal and ambient NO_2_ correlation means that the same key sources contribute to part of their concentrations and perhaps to effects, which were best captured by personal exposure measurements. Among the ICS-only group, personal PM_2.5_ completely confounded ambient PM_2.5_ and personal OC was similarly robust against ambient OC. Again, key sources were likely best captured by personal exposures measurements.

In Supplemental Material (http://www.ehponline.org/docs/2006/9141/suppl.pdf), we present possible reasons for the personal versus ambient pollutant findings in a preliminary assessment of the relationship of personal to indoor and outdoor home exposures.

There was no significant difference in the above associations between subjects taking anti-inflammatory medications versus those who were not. However, Fe_NO_ was associated with 2-day average personal and ambient NO_2_, EC, OC, and PM_2.5_, and ambient PM_10_, among subjects taking ICS, but not in those taking a combination of ICS and antileukotrienes. Consistent differences were found for the last 5 hr of personal PM_2.5_ exposure. This suggests a mixed pattern of susceptibility, because of either treatment regimen or greater ongoing asthma severity. Antileukotrienes are antagonists of type 1 cysteinyl leukotriene receptors. They are believed to have both anti-inflammatory as well as bronchodilator effects on the airways (Lipworth 1999). In a large clinical trial, improvements in most clinical asthma control measures occurred with either fluticasone or montelukast; but outcomes, especially Fe_NO_, improved significantly more with fluticasone than with montelukast treatment (Zeiger et al. 2006). The greater response to air pollutants despite medication suggests that the subjects taking ICS may be more susceptible perhaps as reflected by their persistent asthma. Persistent asthma is a diagnostic indication of the need for anti-inflammatory medication. The addition of antileukotrienes may have achieved additional control sufficient to blunt responses to air pollution exposures. Relevant experimental findings come from two clinical intervention studies showing reduced respiratory responses to air pollutants with montelukast treatment (Gong et al. 2001; Rundell et al. 2005). In addition, antileukotrienes are taken orally once daily, whereas ICS are often prescribed as two to four times per day and they require procedures that are often not followed correctly.

Several other panel studies have found adverse associations between asthma outcomes and criteria air pollutants among subjects taking anti-inflammatory medications (Delfino et al. 2003; Gent et al. 2003; Lewis et al. 2005; Mortimer et al. 2000; Rabinovitch et al. 2006) whereas other panel studies have shown larger associations among subjects not taking any anti-inflammatory medications (Delfino et al. 1998, 2002; Koenig et al. 2003, 2005; Mar et al. 2005). It is conceivable that subjects not taking anti-inflammatory medications who have persistent asthma are more susceptible than comparable subjects who take sufficient controller medications. Further investigation is required to determine how the source and composition of particle exposures contribute to such differences in group susceptibility.

These issues underlie two important limitations of our study. First, we are reliant on surrogate markers of pollutant components (EC, OC, and NO_2_) and limited information on sources. Second, the range of individual mean Fe_NO_ overlapped with concentrations seen in healthy individuals, as previously described (Buchvald et al. 2005). This issue is tied to the still unresolved questions about what Fe_NO_ levels fully represent, and what the between-individual determinants of Fe_NO_ levels are. Other perturbations besides inflammation might increase NO release.

## Conclusions

Exhaled NO in schoolchildren with asthma was associated with personal and ambient background exposure to particulate air pollutants and NO_2_. We found differences in association by medication use, suggesting effect modification and pointing to the importance of assessing individual susceptibility. We also conclude that PM associations may be missed when ambient mass-based methods are used alone, which may not be sufficiently representative of causal pollutant components for some populations or individuals. The relatively weak findings for ambient particle mass compared with EC, OC, and NO_2_, suggest that protecting public health may be insufficient using only a particle mass-based standard, as is currently the case under the U.S. National Ambient Air Quality Standards. Supplemental measurements of particle composition and ultrafine particles (Sioutas et al. 2005), preferably on an hourly scale, are needed to better assess the health impact of particulate air pollution.

We found that association of Fe_NO_ with personal and ambient NO_2_ was largely independent of personal and ambient EC and OC fractions of PM_2.5_ in two-pollutant models, suggesting that both ambient and personal NO_2_ represents other causal pollutant components not sufficiently captured by ambient EC or OC in our study regions. In addition, NO_2_ is a potent oxidant, and experimental data support the hypothesis that oxidative stress may be an important mechanism underlying NO_2_ toxicity (Persinger et al. 2002). To address this gap in knowledge on causal components, continued research is needed on toxic air pollutants expected to have adverse respiratory effects based on biologic mechanisms (e.g., oxidative stress responses). There is also need for health effects studies using source-specific as opposed to a species-specific exposure assessments of air pollution (Ebelt et al. 2005). These recommendations are supported by our contrasting results for personal versus ambient air pollution, especially the two-pollutant model showing independence for personal versus ambient EC. The different lag associations ranging from an immediate Fe_NO_ response to the preceding 5 hr of PM_2.5_ to 2-day average PM_2.5_ also suggest different sources and varying biologic mechanisms are at play.

In addition to the relevance of the results presented here to acute asthma exacerbations, repeated insults from air pollutants may lead to chronic adverse effects on childhood asthma. There is evidence that persistent airway inflammation in asthmatics leads to airway remodeling, diminished lung growth, and permanent lung function impairment (Fabbri et al. 1998).

## Figures and Tables

**Table 1:** Study group characteristics, Riverside and Whittier, California, asthma panels.

Subject variable	Data
Age [years, mean (range)]	13.5 (9–18)
Sex [no. (%)]	
Female	14 (31)
Male	31 (69)
Race [no. (%)]	
Hispanic	26 (58)
White	14 (31)
Black	5 (11)
No. (%) with mean percent FEV_1_ < 80%^a^	11 (24)
Asthma exacerbation previous 12 months required hospital admission, ED visit, or clinic visit	
0 times	12 (27)
1–4 times	19 (42)
> 4 times	14 (31)

a Predicted from NHANES III (Hankinson et al. 1999) using 2 months of thrice daily home spirometry, acceptable and reproducible maneuvers.

**Table 2:** Distribution of Fe_NO_ (ppb) by region and medication use.

Variable	Mean ± SD	Median	25–75th percentile	Range
Overall (45 subjects)	25.6 ± 25.1	18.2	10.5–32.0	2.7–154
Region				
Riverside (13 subjects)	16.6 ± 11.1	13.6	8.8–18.8	3.4–48.5
Whittier (32 subjects)	29.4 ± 28.1^a^	20.8	10.5–33.3	2.7–154
Medication group				
No anti-inflammatory medication (14 subjects)	35.9 ± 35.8	23.9	11.8–46.6	5.0–154
Anti-inflammatory medication (31 subjects)	21.1 ± 16.8^b^	17.5	9.9–26.2	2.7–98.4
Anti-inflammatory medication group				
Inhaled corticosteroids (19 subjects)^c^	20.4 ± 19.5	16.0	6.7–22.1	2.7–98.4
Antileukotrienes ± inhaled corticosteroids (12 subjects)^d^	22.4 ± 10.7^e^	19.6	14.5–30.7	5.0–51.4

a Random-effects model *p* = 0.16 for Fe_NO_ difference from Riverside.

b Random-effects model *p* = 0.06 for Fe_NO_ difference from no anti-inflammatory medication.

c Two subjects were also taking inhaled cromolyn.

d Two subjects were taking antileukotrienes only, and 10 were taking antileukotrienes plus inhaled corticosteroids; mean Fe_NO_ for the two subjects taking antileukotriene medications alone was 21.0 ± 9.1 ppb.

e Random-effects model *p* = 0.66 for Fe_NO_ difference from inhaled corticosteroids.

**Table 3:** Daily air measurements stratified by study panel.

	Riverside panel (*n* = 13)					Whittier panel (*n* = 32)				
Exposure	No. (missing)	Mean ± SD	Median	IQR	Min/max	No. (missing)	Mean ± SD	Median	IQR	Min/max
Personal exposure										
24-hr PM_2.5_ (μg/m^3^)	98 (11)	32.78 ± 21.84	28.14	28.41	7.27/98.43	250 (13)	36.2 ± 25.46	29.07	21.87	7.55/197.05
1-hr max PM_2.5_ (μg/m^3^)	95 (14)	97.94 ± 70.29	83.7	86.75	14.9/431.8	238 (25)	93.63 ± 75.19	71.95	67.67	15.8/572.9
8-hr max PM_2.5_ (μg/m^3^)	95 (14)	47.21 ± 30.9	38.5	45.4	8.9/132.1	238 (25)	51.75 ± 36.88	40.15	33.5	8.7/254.1
24-hr PM_2.5_ EC (μg/m^3^)	100 (9)	0.42 ± 0.69	0.34	0.32	0.01/6.94	246 (17)	0.78 ± 1.42	0.47	0.8	0/17.2
24-hr PM_2.5_ OC (μg/m^3^)	100 (9)	5.63 ± 2.59	4.98	3.36	1.94/12.38	251 (12)	6.81 ± 3.45	6.43	3.97	2.18/31.5
24-hr NO_2_ (ppb)	107 (2)	24.26 ± 9.34	24.31	11.69	5.16/47.61	261 (2)	30.89 ± 14.59	28.5	18.7	2.7/105.7
24-hr temperature	106 (3)	79.88 ± 3.75	79.06	6.14	73.13/88.35	261 (2)	76.55 ± 4.89	77.57	6.86	63.22/86.94
Central site (PM; μg/m^3^)										
24-hr PM_2.5_	109 (0)	36.63 ± 23.46	29.26	30.83	9.52/87.22	252 (11)	18 ± 12.14	16.3	8.67	2.77/77.09
24-hr PM_10_	109 (0)	70.82 ± 29.36	65.96	40.38	30.75/154.05	251 (12)	35.73 ± 16.6	34.65	17.97	5.86/105.46
24-hr PM_2.5_ EC	94 (15)	1.61 ± 0.78	1.35	0.91	0.52/3.64	251 (12)	0.71 ± 0.43	0.63	0.44	0.14/2.95
24-hr PM_2.5_ OC	94 (15)	6.88 ± 1.86	6.07	2.19	4.11/11.62	251 (12)	3.93 ± 1.49	3.76	2.04	1.64/8.82
Central site (gases; ppb)										
8-hr max O_3_	109 (0)	76.37 ± 18.47	73.62	18.62	33.38/120.75	263 (0)	40.56 ± 14.12	38.94	18.1	11.06/79.25
8-hr max NO_2_	109 (0)	39.27 ± 14.6	38	16.14	17.75/72.43	256 (7)	35.05 ± 13.41	30	14.63	13.44/96
24-hr NO_2_	109 (0)	27.18 ± 5.56	24.01	12.15	21.29/34.78	263 (0)	28.07 ± 4.83	29.69	6.22	18.81/35.4
8-hr max CO	109 (0)	530.6 ± 369.58	442.86	514.29	0/1342.86	263 (0)	863.85 ± 523.1	700	600	275/2492.86

Abbreviations: max, maximum; min, minimum.

**Table 4:** Exposure correlation matrix (both study panels pooled).

	Personal				Central			
	PM_2.5_	EC	OC	NO_2_	PM_2.5_	EC	OC	NO_2_
24-hr personal PM_2.5_	1.00	0.18**	0.15*	0.33**	0.64**	0.12*	0.21**	0.22**
24-hr personal EC		1.00	0.41**	0.21**	0.00	0.04	−0.01	0.23**
24-hr personal OC			1.00	0.20**	−0.11*	0.03	−0.02	0.21**
24-hr personal NO_2_				1.00	0.12*	0.19**	0.17*	0.46**
24-hr central PM_2.5_					1.00	0.55**	0.66**	0.25**
24-hr central EC						1.00	0.87**	0.70**
24-hr central OC							1.00	0.62**
24-hr central NO_2_								1.00

* *p*-value < 0.05;

** *p*-value < 0.001 from Wald-based tests of Spearman correlation coefficients.

**Table 5:** Mixed-model estimates of the association between personal and central-site air pollutant exposure and Fe_NO_.

	Personal		Central site	
Exposure	Coefficient^a^ (95% CI)	*p*-Value	Coefficient (95% CI)	*p*-Value
PM_2.5_				
Lag 0	0.42 (−0.15 to 0.99)	0.148	0.03 (−0.68 to 0.74)	0.925
Lag 1	0.51 (−0.10 to 1.12)	0.100	0.44 (−0.28 to 1.16)	0.226
2-day MA	1.01 (0.14 to 1.88)	0.024	0.52 (−0.43 to 1.47)	0.287
PM_2.5_ EC				
Lag 0	0.29 (0.10 to 0.48)	0.003	0.10 (−0.65 to 0.85)	0.793
Lag 1	−0.01 (−0.23 to 0.21)	0.898	0.99 (0.27 to 1.71)	0.007
2-day MA	0.72 (0.32 to 1.12)	0.001	1.38 (0.15 to 2.61)	0.027
PM_2.5_ OC				
Lag 0	0.51 (−0.28 to 1.30)	0.207	0.93 (−0.20 to 2.06)	0.104
Lag 1	0.13 (−0.77 to 1.03)	0.768	0.51 (−0.64 to 1.66)	0.386
2-day MA	0.94 (−0.47 to 2.35)	0.190	1.6 (−0.17 to 3.37)	0.077
NO_2_				
Lag 0	0.25 (−0.44 to 0.94)	0.471	0.10 (−0.55 to 0.75)	0.752
Lag 1	0.60 (−0.12 to 1.32)	0.103	0.72 ( 0.08 to 1.36)	0.028
2-day MA	1.63 (0.43 to 2.83)	0.008	1.36 ( 0.39 to 2.33)	0.006

CI, confidence interval. Lag 0: 24-hr average preceding the Fe_NO_ measurement; Lag 1: average for the 25th through 48th hr preceding the Fe_NO_ measurement; 2-day MA: moving average for the 48 hr preceding the Fe_NO_ measurement.

a The expected change in Fe_NO_ associated with one IQR change in each air pollutant level, adjusted for personal temperature, personal relative humidity, and run. IQRs for personal air pollutant measurements were 24 μg/m^3^ for PM_2.5_, 0.6 μg/m^3^ for PM_2.5_ EC, 4.1 μg/m^3^ for PM_2.5_ OC, and 17 ppb for NO2. IQRs for central-site air pollutant measurements were 15 μg/m^3^ for PM_2.5_, 0.8 μg/m^3^ for PM_2.5_ EC, 2.9 μg/m^3^ for PM_2.5_ OC, and 12 ppb for NO_2_.

**Table 6:** Mixed-model estimates of associations between 2-day moving average personal and central-site air pollutant exposures and Fe_NO_ stratified by medication use.

	Not taking anti-inflammatory medications (14 subjects)		Taking anti-inflammatory medications (31 subjects)		Inhaled corticosteroids (19 subjects)^a^		Antileukotrienes ± inhaled corticosteroids (12 subjects)^b^	
Exposure	Coefficient^c^ (95% CI)	*p*-Value	Coefficient (95% CI)	*p*-Value	Coefficient (95% CI)	*p*-Value	Coefficient (95% CI)	*p*-Value
Personal								
PM_2.5_	1.11 (−1.39 to 3.60)	0.380	1.01 (0.19 to 1.84)	0.017	1.58 (0.72 to 2.43)	0.0004	−0.89 (−2.73 to 0.95)*	0.339
PM_2.5_ EC	0.84 (0.08 to 1.60)	0.031	0.71 (0.28 to 1.15)	0.001	0.67 (0.28 to 1.07)	0.001	0.03 (−3.29 to 3.35)	0.984
PM_2.5_ OC	0.88 (−1.62 to 3.38)	0.484	0.87 (−0.79 to 2.53)	0.302	2.47 (0.30 to 4.64)	0.026	0.52 (−1.99 to 3.02)	0.682
NO_2_	0.80 (−3.01 to 4.61)	0.677	1.67 (0.55 to 2.79)	0.004	1.22 (0.04 to 2.40)	0.043	1.73 (−0.70 to 4.16)	0.160
Central site								
PM_2.5_	0.44 (−1.65 to 2.53)	0.677	0.55 (−0.47 to 1.57)	0.290	1.16 (0.11 to 2.20)	0.030	−0.75 (−2.83 to 1.32)*	0.471
PM_10_	0.76 (−1.54 to 3.07)	0.511	0.53 (−0.83 to 1.90)	0.439	1.28 (−0.01 to 2.58)	0.053	−2.10 (−5.33 to 1.12)*	0.196
PM_2.5_ EC	1.02 (−2.55 to 4.60)	0.570	1.42 (0.25 to 2.60)	0.018	1.28 (0.07 to 2.49)	0.038	1.15 (−1.58 to 3.88)	0.403
PM_2.5_ OC	0.36 (−4.07 to 4.79)	0.870	2.05 (0.24 to 3.86)	0.026	1.96 (0.14 to 3.78)	0.035	1.29 (−2.58 to 5.15)	0.508
NO_2_	0.96 (−1.34 to 3.26)	0.410	1.48 (0.47 to 2.50)	0.004	1.32 (0.33 to 2.32)	0.010	0.67 (−1.86 to 3.20)	0.598

a Two subjects were also taking inhaled cromolyn.

b Two subjects were taking antileukotrienes only, and 10 were taking antileukotrienes plus inhaled corticosteroids.

c The expected change in Fe_NO_ associated with a 1-IQR change in each 2-day moving average air pollutant, adjusted for personal temperature, personal relative humidity, and run. IQR for personal air pollutant measurements were 24 μg/m^3^ for PM_2.5_, 0.6 μg/m^3^ for PM_2.5_ EC, 4.1 μg/m^3^ for PM_2.5_ OC, and 17 ppb for NO_2_. IQR for central-site air pollutant measurements were 15 μg/m^3^ for PM_2.5_, 23 μg/m^3^ for PM_10_, 0.8 μg/m^3^ for PM_2.5_ EC, 2.9 μg/m^3^ for PM_2.5_ OC, and 12 ppb for NO_2_.

* *p* < 0.05 for difference with the coefficient estimate for subjects taking inhaled corticosteroids but not antileukotriene medication.
