# Supplementary material for: Personal and Ambient Air Pollution is Associated with Increased Exhaled Nitric Oxide in Children with Asthma
Source: Environ Health Perspect. 2006 Jul 11;114(11):1736–43. doi: 10.1289/ehp.9141 (PMC1665398; doi:10.1289/ehp.9141)
Supplement: Supplemental Figures and Tables [file ehp0114-001736s1.pdf]

Supplemental Material

Assessment of FE_{NO} data

Out of 446 daily pairs of FE_{NO} samples from the 45 subjects, we found 372 pairs (83%) were reliable by our criteria (≤ 3 ppb NO or ≤ 10% difference), and there was no difference in reliability between regions. The regression slope of the reliable sample pairs was 0.98, intercept 0.6 ppb (R^2 = 0.99). For 74 pairs that did not meet the reliability criteria, six subjects dominated the unreliable pairs (4 or more, total 34). The regression slope of the unreliable sample pairs was 0.71, intercept 10.6 ppb (R^2 = 0.58).

Subjects were asked to refrain from performing spirometry, exercise, and food or beverage intake one hour before sample collection. Field technicians recorded any of these subject proscriptions, and recorded whether the quality of the expiratory maneuver was satisfactory, slow, fast or erratic. The expiration was satisfactory if the subject maintained pressure at 19 cm H_2O, equivalent to 100 ml/sec. The collection was done at the same time of day to minimize the possible effects of circadian rhythm on eNO. We tested with mixed models the relationship between the mean of reliable FE_{NO} samples and the quality of maneuver and other putative determinants.

We compared instances where both FE_{NO} maneuvers were satisfactory to 17 instances where one or both maneuvers were slow, fast or erratic. Unsatisfactory maneuvers were nominally associated with lower FE_{NO} measurements (−1.76 ± 1.24 ppb, p = 0.16). Including this variable in air pollutant models did not confound associations between FE_{NO} and air pollutants.

We tested the effect of subjects having performed their spirometry maneuvers within an hour before the FE_{NO} measurements (N = 79), and compared this with other observations. There was no difference in FE_{NO} (difference 0.14 ± 0.84 ppb). We tested the effects on FE_{NO} from not refraining from exercise, food and beverages one hour prior to the FE_{NO} sampling.

For reports of consuming food (N = 5), the mean for acceptable FE_{NO} pairs was lower compared with FE_{NO} when subjects were fully compliant (–3.80 ± 1.62 ppb, $p < 0.02$), contrary to published reports (ATS-ERS 2005). There was no association between beverage use and FE_{NO}. For reports of exercise (N = 8), the mean for acceptable FE_{NO} pairs was also lower (-3.20 ± 1.37 ppb, $p < 0.02$) as expected (ATS-ERS 2005). Controlling for these noncompliance factors did not confound the associations of FE_{NO} with air pollutants.

Indoor NO for person-days of acceptable FE_{NO} averaged 17 ppb (SD 42), but the median was only 4.9 ppb due to high outliers. Most indoor air samples were < 9 ppb (75th percentile). Only 16 indoor air samples exceeded 100 ppb NO. There was no relationship between indoor NO and acceptable FE_{NO} pairs (slope 0.04 ± 0.03, $p = 0.21$) or unacceptable FE_{NO} pairs (slope 0.02 ± 0.04, $p = 0.57$). In sensitivity analyses, indoor NO concentration did not influence associations of air pollutant exposures with FE_{NO}.

Exposure Assessment Substudy:

The contribution to personal exposures by various environments that subjects move through in a typical day is not assessed here. Nevertheless, in the following we present additional data to provide a better understanding of how the home environment may have contributed to differences in findings for personal as compared with central site exposures. We measured indoor and outdoor $PM_{2.5}$, EC and OC in a subsample of 12 homes for an exposure assessment substudy (one home per 10-day period, 9-10 daily measurements each home). The indoor-outdoor home R^2 was 0.48 for OC and 0.62 for EC. A study done in Riverside County in the region of the present study found for 20 nonsmoking homes the indoor-outdoor home R^2 was 0.47 for OC and 0.63 for EC (Na and Cocker, 2005). Outdoor home $PM_{2.5}$, EC and OC in the present study were also well correlated with central site

measurements (R = 0.91, 0.79, and 0.80, respectively). In contrast, we found a lack of correlation of personal with central site EC or OC (Table 3 of manuscript).

To assess the potential degree of spatial variability that could explain this, we tested the relationship between personal versus home EC and OC measurements for the subsample of 12 homes. This involved 15 subjects during their 10-day exposure assessment period. Sibling pairs with asthma were simultaneously monitored in three of the 12 periods. We found a small Spearman rank correlation between indoor and personal OC (0.21, $p < 0.02$) but no correlation between outdoor home and personal OC (0.04, $p = 0.66$). This is not surprising. Landis et al. (2002) found OC measured on quartz filters represented 168 percent of indoor $PM_{2.5}$ mass on Teflon filters, but only 30 percent of outdoor Teflon filter mass. They postulated that it was due to positive artifact from indoor semi-volatile organic species on quartz filters, which was volatilized on the Teflon filters. Cooking can be a primary source. We found the correlation between indoor OC on quartz filters and indoor $PM_{2.5}$ on Teflon filters was high R = 0.76 ($p <$ 0.0001). For EC we found the Spearman rank correlation was 0.29 ($p < 0.004$) between indoor and personal EC, and 0.25 ($p < 0.02$) between outdoor home and personal EC. This suggests local outdoor sources were affecting personal EC exposures. So why was there a lack of correlation between personal and central site EC? This may be partly attributable to the lower precision of the personal monitor as compared with stationary monitors given the lower sampler flow rates and greater potential for filter cassette leaks. Two other factors are important to consider. First, the central sites could not completely represented the microenvironment immediately outside of the home or the schools of the subjects, including pollutant sources such as traffic, because central sites were up to 9 km away from the subject residences. This is supported by the small but significant correlation of personal EC with both indoor and outdoor home EC measurements. Second, personal exposures are dynamic and include in-vehicle and other exposures close to combustion sources.

References

American Thoracic Society (ATS) and European respiratory Society (ERS). 2005. ATS/ERS Recommendations for Standardized Procedures for the Online and Offline Measurement of Exhaled Lower Respiratory Nitric Oxide and Nasal Nitric Oxide, 2005. Am J Respir Crit Care Med 171:912-930.

Landis MS, Norris GA, Williams RW, Weinstein JP. 2001. Personal exposures to PM2.5 mass and trace elements in Baltimore, MD, USA. Atmos Environ 35:6511-6524.

Na K, Cocker DR. 2005. Organic and elemental carbon concentrations in fine particulate matter in residences, schoolrooms, and outdoor air in Mira Loma, California. Atmos Environ 39:3325-3333.
